# Metabolic changes associated with methionine stress sensitivity in MDA-MB-468 breast cancer cells

**DOI:** 10.1186/s40170-016-0148-6

**Published:** 2016-05-02

**Authors:** Stacey L. Borrego, Johannes Fahrmann, Rupsa Datta, Chiara Stringari, Dmitry Grapov, Michael Zeller, Yumay Chen, Ping Wang, Pierre Baldi, Enrico Gratton, Oliver Fiehn, Peter Kaiser

**Affiliations:** Department of Biological Chemistry, University of California, Irvine, Irvine, CA USA; West Coast Metabolomics Center, University of California, Davis, Davis, CA USA; Laboratory for Fluorescence Dynamics, Department of Biomedical Engineering, University of California, Irvine, Irvine, CA USA; CDS Creative Solutions, Ballwin, MO USA; Department of Computer Science, University of California, Irvine, Irvine, CA USA; Institute for Genomics and Bioinformatics, University of California, Irvine, Irvine, CA USA; Biochemistry Department, King Abdulaziz University, Jeddah, Saudi-Arabia; Present Address: University of Texas M.D. Anderson Cancer Center, Houston, TX USA; Present Address: Laboratory for Optics and Biosciences, Ecole polytechnique, CNRS, INSERM, Université Paris-Saclay, 91128 Palaiseau cedex, France; Present Address: CDS Creative Data Solutions, Ballwin, MO USA

**Keywords:** Cancer metabolism, S-adenosylmethionine, SAM, Methionine, Homocysteine, Methionine stress

## Abstract

**Background:**

The majority of cancer cells have a unique metabolic requirement for methionine that is not observed in normal, non-tumorigenic cells. This phenotype is described as “methionine dependence” or “methionine stress sensitivity” in which cancer cells are unable to proliferate when methionine has been replaced with its metabolic precursor, homocysteine, in cell culture growth media. We focus on the metabolic response to methionine stress in the triple negative breast cancer cell line MDA-MB-468 and its methionine insensitive derivative cell line MDA-MB-468res-R8.

**Results:**

Using a variety of techniques including fluorescence lifetime imaging microscopy (FLIM) and extracellular flux assays, we identified a metabolic down-regulation of oxidative phosphorylation in both MDA-MB-468 and MDA-MB-468res-R8 cell types when cultured in homocysteine media. Untargeted metabolomics was performed by way of gas chromatography/time-of-flight mass spectrometry on both cell types cultured in homocysteine media over a period of 2 to 24 h. We determined unique metabolic responses between the two cell lines in specific pathways including methionine salvage, purine/pyrimidine synthesis, and the tricarboxylic acid cycle. Stable isotope tracer studies using deuterium-labeled homocysteine indicated a redirection of homocysteine metabolism toward the transsulfuration pathway and glutathione synthesis. This data corroborates with increased glutathione levels concomitant with increased levels of oxidized glutathione. Redirection of homocysteine flux resulted in reduced generation of methionine from homocysteine particularly in MDA-MB-468 cells. Consequently, synthesis of the important one-carbon donor S-adenosylmethionine (SAM) was decreased, perturbing the SAM to S-adenosylhomocysteine ratio in MDA-MB-468 cells, which is an indicator of the cellular methylation potential.

**Conclusion:**

This study indicates a differential metabolic response between the methionine sensitive MDA-MB-468 cells and the methionine insensitive derivative cell line MDA-MB-468res-R8. Both cell lines appear to experience oxidative stress when methionine was replaced with its metabolic precursor homocysteine, forcing cells to redirect homocysteine metabolism toward the transsulfuration pathway to increase glutathione synthesis. The methionine stress resistant MDA-MB-468res-R8 cells responded to this cellular stress earlier than the methionine stress sensitive MDA-MB468 cells and coped better with metabolic demands. Additionally, it is evident that S-adenosylmethionine metabolism is dependent on methionine availability in cancer cells, which cannot be sufficiently supplied by homocysteine metabolism under these conditions.

## Background

**Fig. 1:**
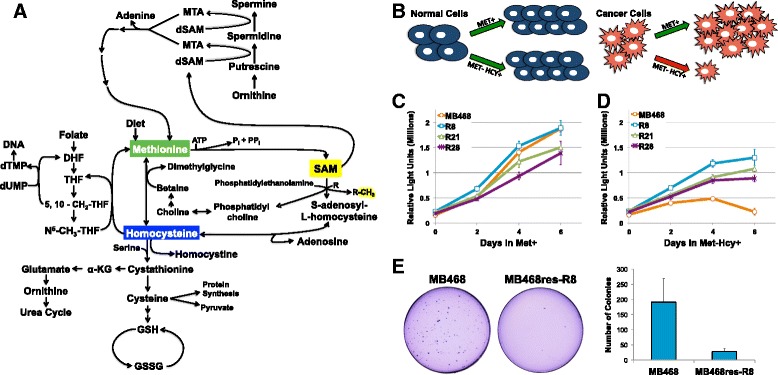
Methionine dependency of cancer cells. **a** Methionine metabolism in mammals: the one carbon cycle is essential to regenerate methionine and S-adenosylmethionine (SAM). SAM is the major methyl donor in the cell and is involved in reactions for phospholipid synthesis, chromatin, RNA, and protein methylation, and one carbon donor pool homeostasis. **b** Illustration depicting the methionine dependency of cancer. Most cancer cells (*red*) do not grow in homocysteine growth medium (Met-Hcy+), whereas normal cells (*blue*) are unaffected. *Green arrows* indicate positive proliferation rates, *red arrows* indicate reduced or no proliferation. **c** MB468 and MB468res cells proliferate in methionine growth media (Met+) at similar rates. **d** But only the resistant cells (MB468res: R8, R21, R28) maintain proliferation in Met-Hcy+ media. Proliferation rates were quantified by luminescent cell viability assay. *Error bars* represent standard deviation. **e** Methionine stress resistant clone MB468res-R8 forms fewer colonies in soft agar than the parental MB468 cell line. Cells were plated in 0.3 % agar, cultured for 30 days, and stained with crystal violet. Colony values are the average of three independent experiments. *Error bars* indicate standard deviation

Reprogrammed metabolism in cancer has gained greater attention in recent years, beyond the well-known Warburg effect [1]. While mechanistic insights into the metabolic changes in cancer are limited, the importance of methionine metabolism in cancer cell proliferation has been known for over 50 years (Fig. 1a) [2]. Early studies describe a unique metabolic requirement of cancer for methionine known as the “methionine dependence phenotype.” The vast majority of malignant cells experience methionine dependence and cannot proliferate in growth media in which methionine is replaced by its metabolic precursor, homocysteine. In contrast, most normal non-tumorigenic cells are unaffected by culturing in homocysteine media (Fig. 1b) [3].

Methionine is an essential amino acid necessary for normal growth and cell function. It contributes to protein synthesis and is the precursor to S-adenosylmethionine (SAM), the principal methyl donor in the cell. SAM is a versatile molecule required for methylation of DNA, RNA, proteins, and lipids by a variety of methyltransferases. In addition, SAM is critical for the formation of 1-methylnicotinamide, a primary factor involved in stem cell pluripotency [4], polyamine biosynthesis [5], and the methionine salvage pathway [6]. As SAM donates its activated methyl group in methylation reactions, it is converted to S-adenosylhomocysteine, which is further hydrolyzed to homocysteine in a reversible reaction (Fig. 1a) [7]. Homocysteine is a junction metabolite, and its metabolism can be either directed toward the remethylation pathway to regenerate methionine by receiving a methyl group from betaine or N_5_-methyltetrahydrofolic acid or toward cysteine and glutathione synthesis in the transsulfuration pathway [8]. Homocysteine inhabits a critical position where, depending on demand, metabolic flux can be redirected to increase methylation potential or produce antioxidants.

Although initial studies suggested methionine limitation to be responsible for the “methionine dependence phenotype,” limited availability of SAM is the actual culprit. Work by Coalson and colleagues has shown that methionine dependent cells endogenously synthesize methionine at normal levels in homocysteine media (Met-Hcy+) but show reduced SAM synthesis [9]. Accordingly, by supplementing homocysteine growth medium with SAM, cell proliferation of methionine-dependent breast cancer cells can be restored, suggesting SAM limitation as the cause for methionine dependence [10]. Furthermore, SAM limitation induced by knockdown of methionine adenosyltransferase (MAT), the enzyme responsible for synthesis of SAM from methionine and ATP, mimics the cell cycle arrest induced by replacing methionine with homocysteine in the growth media [10, 11]. The specific cell cycle arrest in the G_1_ phase induced by homocysteine medium or MAT knockdown is reminiscent of an evolutionary conserved metabolite responsive cell cycle checkpoint first described in yeast. This “SAM-checkpoint” was proposed to protect cellular integrity and maintain epigenetic stability as it halts cell cycle progression before intracellular SAM concentrations get too low to support the various methylation reactions necessary for normal cell physiology. Cancer cells have a highly responsive SAM-checkpoint, likely because of their higher demand on SAM [10]. Details of why cancer cells depend on high levels of SAM remain to be discovered, but their increased proliferation rate requires constant duplication of chromatin methylation marks, methylation of RNAs, and SAM-dependent synthesis of membrane lipids. In addition, many cancer cells are characterized by hyperactive polyamine synthesis [12], which consumes SAM. A decarboxylated form of SAM reacts with the polyamine putrescine to form spermidine and spermine, generating the by-product 5′-deoxy-5′-methylthioadenosine (MTA). Further processing of MTA generates adenine and replenishes the methionine pool [13]. Although polyamine synthesis clearly contributes to SAM usage in cancer cells and modified polyamines, specifically diacetylspermine, have been shown as clear biomarkers in lung [14] and colon cancer [15], SAM limitation or growth in homocysteine media do not imitate effects of polyamine depletion and cannot be rescued by spermidine supplementation [11, 16].

While some progress has been made by linking methionine dependency of cancer to SAM limitation and induction of a specific cell cycle arrest, our understanding of this striking metabolic dependence of cancer remains minimal. Here, we characterized the dynamics of metabolic changes in breast cancer cells when they are shifted to homocysteine-based growth media, using untargeted metabolomics as well as stable isotope-labeled tracer studies to follow the metabolic fate of homocysteine.

## Methods

### Cell lines and growth conditions

MDA-MB-468 were maintained in DMEM (Sigma-Aldrich, D0422) supplemented with 10 % dialyzed FBS (Gemini Bio-Products), 1.5 μM cyanocobalmin (vitamin B12), 4 mM l-glutamine, 100 μM l-cysteine (Fisher Scientific), and 100 μM l-methionine (Sigma-Aldrich). In the case of methionine-free media, 370 μM DL-homocysteine (Sigma-Aldrich) or 370 μM DL-homocysteine-^2^H_4_ (13C Molecular, 12714-158) was added in the absence of methionine.

Resistant cell lines were isolated as described in Hoffman et al. [17]. Briefly, MDA-MB-468 resistant clones were isolated after prolonged culturing in methionine-free media. The majority of MDA-MB-468 cells detach; however, resistant clones begin to appear after 2 weeks. Clones were isolated, and proliferation rates were measured using CellTiter-Glo luminescent cell viability assay (Promega).

Both MD468 and MB468res-R8 cell lines were tested for authentication via STR profiling in January 2016 by Genetica DNA Laboratories (a LabCorp brand; Burlington, NC) using the commercially available PowerPlex® 16HS amplification kit (Promega Corporation) and GeneMapper ID v3.2.1 software (Applied Biosystems). Authentication of each cell line was confirmed by a 100 % match to the reference STR profile of MDA-MB-468 (ATCC HTB-132) cells from ATCC.

### Anchorage independence assay

To detect anchorage independence of transformed cells, MDA-MB-468 and resistant cell lines were cultured in soft agar for 30 days. Each assay was conducted in a 35-mm Petri dish with a 0.5 % agar base layer and 0.35 % agar top layer prepared using low-gelling-temperature agarose (Sigma, A9045) in methionine growth media. The top layer of each assay contained 5000 cells except for control plates. Assays were maintained at 37 °C in a humidified incubator, and 0.5 ml methionine growth media was added to each plate twice weekly. Colonies were stained with 0.005 % crystal violet for 1 h, washed once with PBS, and manually counted.

### Preparing cells for metabolite extraction

Cells were harvested by first washing attached cells with 10 ml PBS before adding 0.5 % trypsin (Life Technologies, 25300-054) for 10 min and incubating at 37 °C. Cells were collected in complete DMEM (4 °C) without methionine or homocysteine and centrifuged (300×*g*, 5 min, 4 °C); from this point on, cells were kept on ice during all processing steps. Cell pellets were washed with 10 ml PBS (4 °C) and counted. The appropriate number of cells was aliquoted and centrifuged (300×*g*, 5 min, 4 °C). Cell pellets were resuspended in 1 ml PBS (4 °C), transferred to a pre-weighed microcentrifuge tube, and centrifuged (300×*g*, 5 min, 4 °C). PBS was aspirated, and cell pellets were weighed before flash freezing in liquid nitrogen. Samples were stored at −80 °C until shipped on dry ice. This protocol was performed as described in T.W.-M. Fan et al. [18].

### Immunoblots

MDA-MB-468 and MDA-MB-468res-R8 cells were cultured in methionine or homocysteine media for 24, 48, 72, and 96 h. Lysates were prepared in 8 M urea buffer (8 M urea, 200 mM NaCl, 0.2 % SDS, 100 mM Tris (pH 7.5), 1 mM PMSF, 1 ng/ml aprotinin) and resolved by 10 % SDS-PAGE. Protein levels were detected by immunoblot using MAT2A (Abcam, ab77471) and MAT2B (Abcam, ab86506) antibodies.

### FLIM acquisition and phasor data analysis

MDA-MB-468 and MDA-MB-468res-R8 cells were cultured in methionine or homocysteine media. To inhibit oxidative phosphorylation, cells were treated with 4 mM potassium cyanide (KCN) for 5 min before imaging.

Fluorescence lifetime imaging microscopy (FLIM) was performed on Zeiss LSM 710 microscope (Carl Zeiss, Jena, Germany) using a ×40 water immersion objective, 1.2 N.A. (Carl Zeiss, Oberkochen, Germany) coupled with two-photon excitation of 740 nm (titanium:sapphire MaiTai laser from Spectra-Physics, Mountain View, CA). Image scan speed was 25.21 μs/pixel, and image size is 256 × 256 pixels. The emission signal was collected using a 460/80-nm bandpass filter and photomultiplier tube (H7422P-40, Hamamatsu, Japan) was used for detection. FLIM data was acquired using A320 FastFLIM FLIMbox (ISS, Champaign, IL). SimFCS software, developed at the Laboratory for Fluorescence Dynamics (LFD, UC Irvine), was employed for both FLIM data acquisition and analysis. FLIM-phasor approach was used for phasor analysis, as described previously [19, 20]. Briefly, lifetime information from every pixel of the image is transformed into a phasor and plotted on a phasor plot. This can be mapped back onto the image to create the FLIM map.

### Extracellular flux (XF) analyses

All XF analyses were performed on the Seahorse Bioscience XF24 Extracellular Flux Analyzer (Seahorse Bioscience, North Billerica, MA) using the manufacturer’s protocol. Cells were plated in 24-well Seahorse XF-24 assay plates at a density of 90,000 cells per well in Met+ media (as described above). After 6 h, eight wells were washed with pre-warmed PBS before Met+ media and Met-Hcy+ media were added in four wells each. This process was repeated for the 8, 4, and 2 h time points. One hour before metabolic flux analysis, cells were washed once with un-buffered, serum-free media (Caisson Labs, DMP39-10XLT) containing 100 uM methionine or 370 μM DL-homocysteine and incubated with un-buffered media at 37 °C in a non-CO_2_ incubator for 1 h. Four baseline oxygen consumption rate (OCR) and extracellular acidification rate (ECAR) measurements were taken before sequential injection of the following mitochondrial inhibitors: oligomycin (1 μg/ml), carbonyl cyanide-4-phenylhydrazone (FCCP) (0.4 μM), and rotenone (0.1 μM). Four measurements were taken after the addition of each inhibitor, and OCR and ECAR values were automatically calculated and recorded by the Seahorse XF-24 software. Basal respiration was calculated by averaging the four measurements of OCR before injection of inhibitors. Basal ECAR values were calculated in the same manner. Spare respiration capacity was calculated by subtracting basal OCR values from OCR values after FCCP treatment.

### Metabolomics analysis

The MiniX database [21] was used as a Laboratory Information Management System (LIMS) and for sample randomization prior to all analytical procedures.

#### GCTOFMS analysis

For analysis of primary metabolism, cell lysates, stored at −80 °C, were thawed on ice, extracted, and derivatized and metabolite levels were quantified by gas chromatography time-of-flight (GC-TOF) mass spectrometry (MS) as previously described [22]. Acquired spectra were further processed using the BinBase database [21, 23]. Briefly, output results [24] were filtered based on multiple parameters to exclude noisy or inconsistent peaks. All entries in BinBase were matched against the Fiehn mass spectral library of 1200 authentic metabolite spectra using retention index and mass spectrum information or the NIST14 commercial library. Data, reported as quantitative ion peak heights, were normalized by the sum intensity of all annotated metabolites (Eq. 1) and used for further statistical analysis.

1 $$ {\mathrm{metabolite}}_{\mathrm{ij},\mathrm{normalized}} = {\mathrm{metabolite}}_{\mathrm{ij},\ \mathrm{raw}}/{\mathrm{mTIC}}_{\mathrm{j}}*\ \mathrm{mTIC}\ \mathrm{average} $$

#### HILIC-UHPLC-qTOFMS analysis

For analysis of biogenic amines, cell lysates, stored at −80 °C, were thawed on ice and extracted with ice-cold “degassed” 3:3:2 acetonitrile/isopropanol/ultra-pure water. Supernatant-containing extracted metabolites were dried to completeness under reduced pressure and resuspended in 60 μL of 80:20 ACN/H2O containing an internal Val-Tyr-Val (Sigma-Aldrich). Resuspended samples were analyzed on an Agilent 1290A Infinity Ultra High Performance Liquid Chromatography system with an Agilent Accurate Mass-6550-QTOF mass spectrometer. The column (45 °C) was a Waters Acquity UPLC BEH (150-mm length × 2.1-mm internal diameter; 1.7-μM particles) coupled with a Waters Acquity VanGuard BEH C18 Pre-column (50-mm length × 2.1-mm internal diameter; 1.7-μM particles). The solvent system included (A) 100 % water (LCMS grade) containing 10 mM ammonium formate and 0.125 % formic acid and (B) 95:5 *v*/*v* acetonitrile to water containing 10 mM ammonium formate and 0.125 % formic acid. The gradient started from 0 min 100 % (B), 0–2 min 100 % (B), 2–7.7 min 70 % (B), 7.7–9.5 min 40 % (B), 9.5–10.25 min 30 % (B), 10.25–12.75 min 100 % (B), and 12.75–16.75 min 100 % (B). The flow rate was 0.4 mL/min and with an injection volume of 5 μL. ESI capillary voltage was +3.5 kV with collision energies of 20 eV MSMS collection in positive acquisition mode. Data was collected at a mass range of m/z 60–1700 Da with a spectral acquisition speed of 4 spectra per second.

Data was processed using MZmine 2.10 software. Metabolites were identified by searching against a precursor accurate mass and retention time library in conjunction with matching tandem mass spectra against the LipidBlast virtual MS/MS database [25]. Data are reported as peak heights for the quantification ion (m/z) at the specific retention time for each annotated and unknown metabolite.

### Stable isotope tracer studies

#### Evaluation of methionine, homocysteine, and cystathionine enrichment

Cells were extracted with ice-cold 1 ml of degassed 3:3:2 acetonitrile/isopropanol/ultra-pure water, the supernatant removed, and solvents evaporated to dryness under reduced pressure. To remove membrane lipids and triacylglycerides, dried samples were reconstituted with acetonitrile/water (1:1), decanted, and taken to dryness under reduced pressure. Samples were derivatized with methoxyamine hydrochloride in pyridine and subsequently by MTBSTFA (Sigma-Aldrich) and analyzed by gas chromatography mass spectrometry.

An Agilent 7890A gas chromatograph (Santa Clara, CA) was used with a 30 m × 0.25-mm i.d. (internal diameter) × 0.25 μM HP-5 MS Column (Agilent J&W GC Columns). An Agilent 7693 auto-sampler was used to eliminate cross-contamination during GCMS analysis. One microliter (1 μl) of sample was injected at 60 °C (ramped to 250 °C) in splitless mode with a 30-s purge time. The chromatographic gradient consisted of a constant flow of 1 mL/min, ramping the oven temperature from 60 to 350 °C over 37 min. Mass spectrometry was done using an Agilent 5977A MSD spectrometer, 290 °C transfer line temperature, electron ionization at −70 eV, and an ion source temperate of 230 °C. Mass spectra were acquired at 1555 V at m/z 50–600 with 2.7 spectra/s.

Acquired spectra were converted to netCDF files using vendor (Agilent) software and submitted for non-targeted enrichment analysis using the Non-targeted Fate Detection Software version 1.1 [26]. Spectrum of enriched peaks was manually compared against reference spectrum-derived authentic standard and from parallel experiments using non-labeled homocysteine.

Enrichment due to presence of deuterium was validated using a secondary independent mass isotopomer distribution (MID) analyzer developed at the West Coast Metabolomics Center. MID calculations were determined using a modified least-squares linear regression matrix modeled after Jennings et al. [27]; corrected for natural abundance; and reduced isotopic probability with increasing deuterium enrichment. MID values are reported as fractional proportions (1 = 100 % enriched) that the respective mass isotopologue contributes to the overall abundance of the respective analyte.

#### Evaluation of SAM and SAH enrichment

SAM and S-adenosylhomocysteine (SAH) levels were determined by hydrophilic interaction liquid chromatography ultra high pressure liquid chromatography accurate mass quadrupole time-of-flight mass spectrometry (HILIC-UHPLC-qTOFMS) as described above. Enrichment due to presence of deuterium was determined using the MID analyzer as described above. Spectrum of enriched peaks was manually compared against reference spectrum-derived authentic standard and from parallel experiments using non-labeled homocysteine.

## Results and discussion

### A methionine-dependent and methionine-independent breast cancer cell line pair

The majority of malignant cells exhibit the methionine-dependent phenotype as indicated by cell cycle arrest and apoptosis when cultured in media where methionine (methionine media (Met+)) is replaced with the immediate metabolic precursor homocysteine (homocysteine media, Met-HCY+) [3, 28, 29]. Although methionine dependence is specific for cancer cells and non-tumorigenic cells proliferate well in homocysteine medium, long-term culturing of malignant cell lines in Met-Hcy+ can select methionine-independent, methionine stress resistant clones [10, 30]. The data presented in this study focuses on the triple negative breast cancer cell line MDA-MB-468 (shortened to MB468) due to our ability to derive multiple methionine stress resistant clones (MB468res) as controls [10]. These methionine stress resistant cells propagate at similar rates as the parental MB468 cell line when cultured in methionine growth medium; however, MB468res cells continue to proliferate in Met-Hcy+ medium unlike MB468 cells (Fig. 1c, d). Spontaneous reversion to methionine independence has been associated with loss of transformation-specific properties such as anchorage independence [31]. We therefore tested whether phenotypes associated with tumorigenicity are also lost in MB468res cells. When cultured in soft agar with Met+ growth media, MB468 cells efficiently formed colonies consistent with their tumorigenic phenotype. In contrast, MB468res-R8 cells formed only few or very small colonies (Fig. 1e), indicating a loss of the transformation phenotype. Similar results were obtained with the MB468res-R21 and R28 lines. However, whereas R8 and R21 did not form colonies, the R28 line formed numerous tiny colonies scarcely visible with the naked eye. We continued our studies with the MB468res-R8 cell line as their proliferation rate in Met+ medium is closest to the parental MB468 cell line, which eliminates potential effects on metabolite demand due to growth rate differences. MB468res-R8 cells provide an excellent control for our investigation of methionine dependence because cell morphology, proliferation rate, growth medium requirements, and genetic background resemble those of methionine stress sensitive MB468 cells. The molecular mechanism of reversion to methionine independence is unknown. Reversion is an extremely rare event and stable for several generations. However, after several generations, MB468res-R8 cultures can revert back to methionine dependence, suggesting epigenetic mechanisms behind this phenomenon.

### Homocysteine media induces a metabolic down-regulation of oxidative phosphorylation

**Fig. 2:**
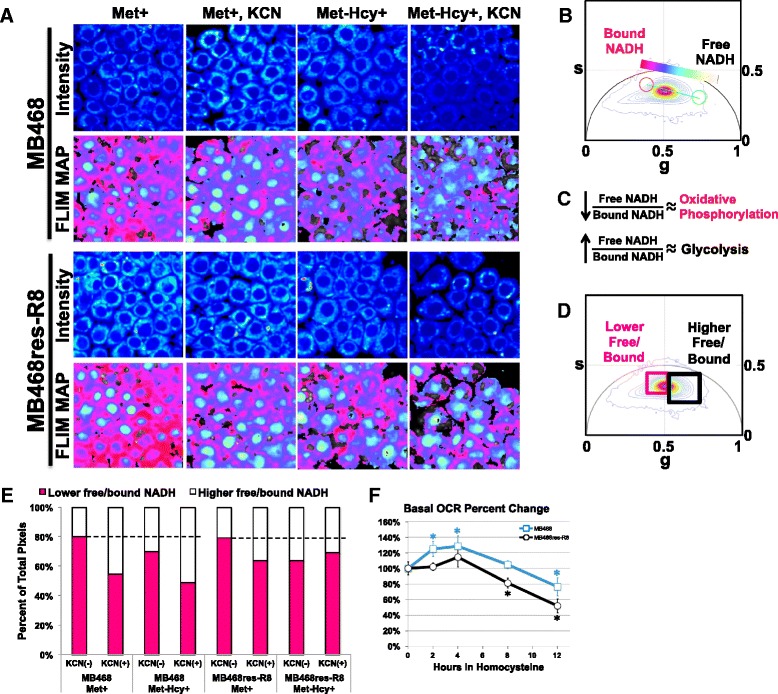
Homocysteine media induces a metabolic shift from oxidative phosphorylation to glycolysis. **a** MB468 and MB468res-R8 cells cultured in Met+ or Met-Hcy+ growth media for 48 h with or without 4 mM KCN were imaged using two-photon microscopy at 740 nm. NADH free/bound fluorescence lifetime imaging microscopy (FLIM) maps of cells are shown using the color spectrum in the FLIM phasor histogram (**b**). **c** The phasor histogram represents relative fractions of free NADH (*cyan-white*) and protein-bound NADH (*red-purple*). **d** Cluster analysis using the phasor histogram to separate signals of high free/bound (*black square*) and low free/bound (*pink square*) NADH ratios. **e** Quantitation of NADH ratios from cells in **a** is shown. Two fields for each sample with a minimum of 15 cells per field were analyzed by cluster analysis and normalized to total pixels measured. The *black dotted line* indicates the average percentage of lower free/bound NADH ratios of MB468 (80 %) and MB468res-R8 (79 %) cells cultured in in Met+, KCN(−). **f** MB468 and MB468res-R8 cells cultured in Met+ media for 12 h or Met-Hcy+ media for 2, 4, 8, and 12 h were analyzed by Seahorse Bioscience XF analyzer. MB468 (*blue line* with *squares*) and MB468res-R8 (*black line* with *circles*) basal oxygen consumption rates (OCR) are shown normalized to the Met+-treated sample. *Error bars* represent normalized standard deviation, *n* = 4 replicates; statistical differences between Met+ media and Met-Hcy+ media treatments are indicated by *asterisk* where *p* ≤ 5.0 × 10^−7^

To monitor metabolic states in MB468 cells and their methionine-independent derivatives, MB468res-R8, we employed FLIM. Using two-photon excitation at 740 nm, we measured the intrinsically fluorescent molecule-reduced nicotinamide adenine dinucleotide (NADH) in live cells (Fig. 2a). NADH is a powerful biomarker for the metabolic state of the cell as it is the principal electron donor in glycolysis and electron acceptor in oxidative phosphorylation [32]. Phasor analysis of NADH FLIM data gives spatial distribution of free to protein-bound NADH [19, 20, 33] (Fig. 2b). Importantly, FLIM analysis is different from the intensity ratio at two different emission wavelengths, which is commonly used to correlate with metabolism. In the FLIM analysis, only NADH in the bound and free form is measured by proper selection of excitation and emission wavelengths. More importantly, the FLIM technique is based on fluorescence lifetime, which is independent of the total concentration of NADH, contrary to the common dual wavelength emission ratio. In our analysis, we determine directly the free to bound (to proteins) ratio. Protein-bound NADH is characterized by longer fluorescence lifetimes, influenced by binding to various enzymes. In contrast, shorter fluorescence lifetimes of NADH indicate a higher fraction of NADH that is not bound to proteins [34]. By measuring the NADH phasor distribution along the metabolic trajectory on the phasor plot, we can calculate the free/bound NADH ratio [35]. A series of previous experiments have shown that low free/bound NADH ratios are characteristic for cells actively engaged in oxidative phosphorylation, whereas higher free/bound ratios indicate increased dependence on glycolysis for energy production (Fig. 2c) [19, 20, 36].

We compared fluorescence lifetimes of NADH from both MB468 and MB468res-R8 cells cultured in Met+ and Met-Hcy+ media. We used phasor cluster analysis to quantify the signals of higher and lower free/bound NADH ratios of both cell lines (Fig. 2d). In Met+ media, both cell lines had similar profiles with approximately 80 % of the signal representing a lower free/bound ratio providing a baseline for oxidative phosphorylation activity in these cell types (Fig. 2e). After 48 h, cells were transferred to Met-Hcy+ media; 70 % of the MB468 and 64 % of the MB468res-R8 signal represented the lower free/bound NADH ratio and thus a reduction in cellular respiration. Importantly, the addition of potassium cyanide, a potent inhibitor of mitochondrial respiration, resulted in a decrease in the signal of the lower free/bound ratio, similar to culturing in Met-Hcy+ media alone (Fig. 2a, e). The similar metabolic trajectories of potassium cyanide-treated cells and Met-Hcy+ cultures suggest that methionine stress may affect mitochondrial function or force cells to reduce oxidative phosphorylation.

We further analyzed mitochondrial function using an extracellular flux (XF) analyzer, which measures OCR as an indicator for mitochondrial respiration. Both MB468 and MB468res-R8 cells were cultured in either Met+ media for 12 h or Met-Hcy+ media for 2, 4, 8, and 12 h prior to measuring basal levels of OCR (Fig. 2f). Within 4 h in Met-Hcy+ media, both MB468 and MB468res-R8 cell lines respond by transiently increasing OCR. However, after 8 h, both cell lines showed decreased respiration, which continued to decline. Interestingly, we observed the MB468res-R8 cells to have a faster decline in respiration as compared to MB468 cells. In additional analyses with the XF analyzer, spare respiration capacity was measured in MB468 and MB468res-R8 cells cultured in Met+ media and treated with the electron transport chain accelerator FCCP. MB468 cells can maximize OCR 1.9 times that of basal levels when stressed with FCCP, whereas MB468res-R8 cells have a maximum 1.5-fold increase of OCR under the same conditions. We hypothesize that the faster decline in mitochondrial respiration in Met-Hcy+ media (Fig. 2f) and the reduced effect of KCN in the FLIM analysis (Fig. 2e) as compared to MB468 cells may be due to an innate lower capacity for respiration. With a smaller reserved respiration capacity, MB468res-R8 cells may feel the effects of dysfunctional mitochondria sooner but may not be as reliant on oxidative phosphorylation resulting in improved tolerance of metabolic stress as compared to MB468 cells. Regardless of these differences, the general response of both cell lines to Met-Hcy+ media and KCN is comparable, indicating reduced mitochondrial respiration during culturing in Met-Hcy+ media as determined by both FLIM and XF analyses.

### Metabolite changes associated with culturing in homocysteine media

**Fig. 3:**
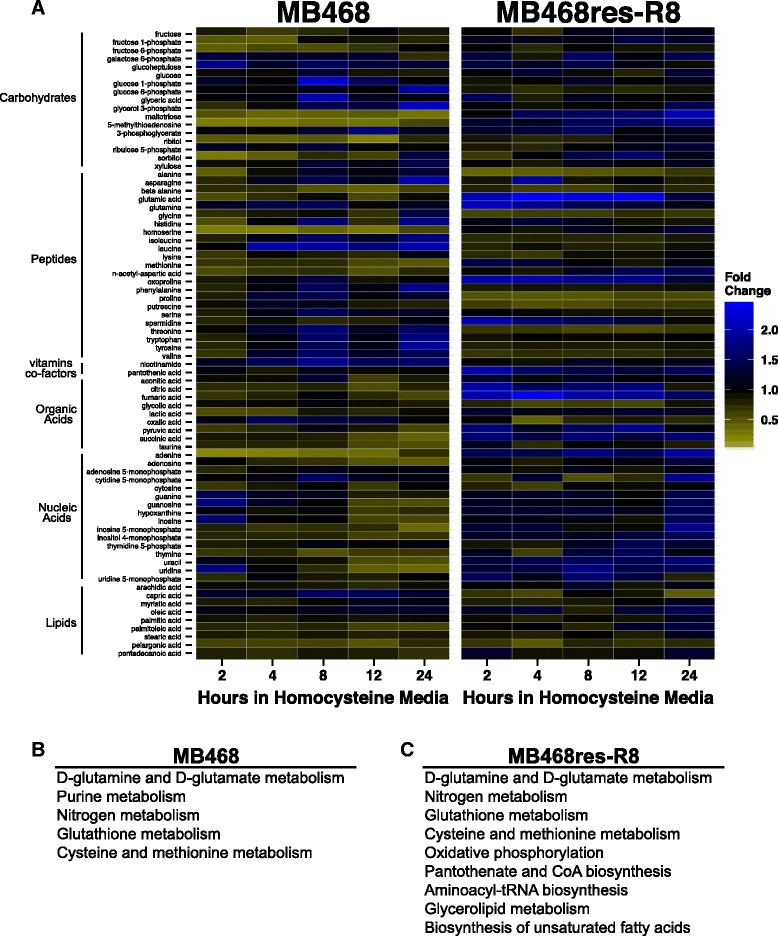
Homocysteine media induces a metabolic response in MB468 and MB468res-R8 cells. MB468 and MB468res-R8 cells were cultured in Met+ or Met-Hcy+ media over the course of 24 h and analyzed by untargeted UHPLC- and GCMS-based methodologies (see “Methods” section). **a** Heatmaps representing metabolites measured in both MB468 and MB468res-R8 cells. Metabolites are color filled based on fold change to the Met+, time-zero sample. *Yellow* indicates a decrease in metabolite abundance and *blue* indicates an increase as compared to time-zero control. **b**, **c** Pathways enriched within 4 h of culturing in Met-Hcy+ media. Metabolites with significantly different abundance at 2 and 4 h as compared to time zero were determined using the Bayesian *t* test, Cyber-T [37, 38], with a Benjamini-Hochberg *p* ≤ 0.05. Enriched pathways were determined using the web server Metabolite Biological Role (MBRole) with a raw *p* value cutoff at *p* ≤ 0.1 [39]

FLIM and XF analyses indicated that cells change their metabolism in response to homocysteine medium. These results prompted us to investigate the immediate metabolic response to homocysteine media. Both MB468 and MB468re-R8 were cultured in Met+ media or transitioned to Met-Hcy+ media for 2, 4, 8, 12, and 24 h, and metabolites were analyzed by an untargeted metabolomics approach using gas chromatography/time-of-flight mass spectrometry (Fig. 3a). Immediate changes in select metabolites are observed for both cell lines, albeit with distinct trends. Interpretation of differential changes of metabolite concentrations between the two cell lines at the later time points is complicated by the fact that MB468 cells respond with cell cycle arrest whereas MB468re-R8 cells continue to proliferate. It is thus difficult to distinguish between changes due to homocysteine growth media and changes caused by the block in proliferation. However, metabolite concentration changes at the early time points cannot be indirect consequences of cell cycle arrest as the time frame for cell cycle arrest is substantially longer [10]. Interestingly, the general trends of metabolite concentration changes between the cell lines seem to project into different directions. In MB468 cells, several metabolites responded within 4 h with a general decrease in abundance as compared to Met+-cultured cells. In contrast, the majority of metabolites in MB468res-R8 cells that responded within 4 h increased in their abundance. Pathway analysis using the web server Metabolite Biological Role showed that within 4 h of culturing in Met-Hcy+ media, fewer pathways are affected in MB468 cells as compared to MB468res-R8 (Fig. 3b, c) [37–39].

#### Metabolites connected to methionine

**Fig. 4:**
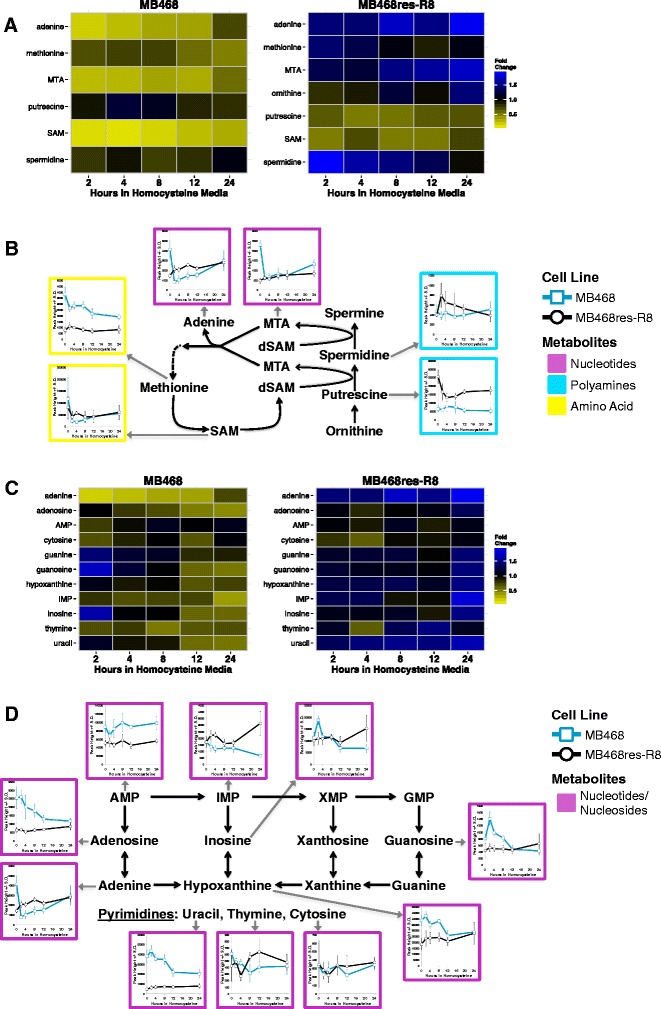
Metabolite changes linked to methionine and nucleoside metabolism. **a** MB468 and MB468res-R8 cells were cultured in Met+ or Met-Hcy+ media over the course of 24 h and analyzed by untargeted GCTOFMS and HILIC-UHPLC-qTOFMS-based metabolomics. Heatmaps represent fold changes of metabolites in the purine salvage as compared to the Met+, time-zero sample. Metabolites are color filled by fold change: *yellow* indicates a decrease in metabolite abundance and *blue* indicates an increase as compared to time-zero control. **b** Schematic of polyamine synthesis and methionine regeneration by way of the methionine salvage pathway. Metabolites with *gray arrows* are pointing to line graphs of the corresponding metabolite abundances in MB468 (*blue line* with *squares*) and MB468res-R8 (*black line* with *circles*) cells. Peak height reflecting relative metabolite abundance is marked on the *y*-axis and hours in homocysteine are marked on the *x*-axis. *Error bars* represent standard deviation. **c** Heatmaps represent fold changes of metabolites involved in purine/pyrimidine synthesis. Color fill is the same as **a**. **d** Schematic of purine synthesis and pyrimidines with corresponding metabolite abundances, same as **b**

We next looked at individual metabolic pathways in more detail. Changes in metabolite levels associated with methionine stress are evident in both MB468 and MB468res-R8 cell lines as early as 2 h after switching to Met-Hcy+ media. Several metabolite concentration changes occurred in both the methionine-dependent (MB468) and methionine-independent (MB468res-R8) cell lines and reflect a general response to homocysteine becoming the primary source of sulfur amino acids. Metabolite responses with distinct patterns in the two cell lines were particularly evident for metabolites connected to the methionine salvage pathway and purine/pyrimidine synthesis (Fig. 4). We found a striking response in levels of 5′-deoxy-5′-methylthioadenosine (MTA) and adenine, the two SAM-derived metabolites in the methionine salvage pathway. Both MTA and adenine decreased rapidly in MB468 cells but not in MB468res-R8 (Fig. 4a). MTA is the by-product of polyamine synthesis, and adenine is released as MTA is recycled into methionine (Fig. 4b). MTA and adenine concentrations decreased to about one fifth of the starting concentration within 2 h after MB468 cells were shifted to Met-Hcy+ medium and recovered somewhat to about 60 % of starting levels after 24 h. Surprisingly, even though the rapid and dramatic reduction in MTA and adenine levels suggest repression of polyamine synthesis, the levels of putrescine and spermidine remained largely unchanged over the time course (Fig. 4b). It is possible that the amount of polyamines synthesized over the 24 h period we monitored does not significantly contribute to the total polyamine pool in cells. However, the decrease in MTA production clearly reflects a physiological response of MB468 cells to methionine stress and suggests that cells attempt to preserve SAM concentrations by repression of polyamine synthesis. In contrast, MB468res-R8 cells appear to up-regulate polyamine synthesis indicated by doubling of spermidine abundance within 2 h and a correction to starting levels after 24 h. MTA and adenine levels show a similar increase in abundance over 2 h and continue to increase after 24 h to almost twice the starting levels.

The differences in the levels of MTA may contribute to the difference in methionine levels between the two cell lines. MB468 cells have a slight decrease in methionine starting at 2 h, staying around 0.7-fold abundance until 24 h when the levels decrease to half the starting quantity (Fig. 4b). MB468res-R8 cells increase in methionine levels as early as 2 h but return to normal after 8 h. It is important to note that the MB468res-R8 cells continue to proliferate in Met-Hcy+ media unlike the MB468 cells, which may affect the use of metabolites in each cell line after 24 h, the approximate doubling time of these cells. However, the early changes we observed can be attributed to a metabolic response to the switch from methionine to homocysteine in the growth medium.

#### Purines and pyrimidines

Both adenine and adenosine are by-products of the methionine salvage and remethylation pathways and are essential components in purine synthesis. With few exceptions, changes in purine as well as pyrimidine concentrations were not evident until 12 to 24 h after cells were shifted (Fig. 4c). In MB468res-R8 cells, only adenine increased to approximately 1.5-fold of the starting quantity within 2 h. After 12 and 24 h, inosine, inosine monophosphate, and hypoxanthine were significantly increased (Fig. 4d). In contrast, MB468 cells respond to Met-Hcy+ medium with a delayed decrease in inosine, guanine, and guanosine, as well as an immediate steep decrease in adenine levels as described above. Unlike the overall increase in purines in MB468res-R8 cells, many purines in MB468 cells decrease to half the starting quantity by 24 h. Interestingly, the changes in components of the purine synthesis pathway are not observed for the pyrimidines thymine and cytosine. In both MB468 and MB468res-R8 cells, thymine and cytosine abundance indicate a minimal response to methionine stress. Uracil, the pyrimidine derivative found in RNA, showed a comparable trend to the purine response in both cell lines as levels decrease in MB468 cells and increase in MB468res-R8 cells over the course of 24 h (Fig. 4d).

#### Glycolysis and citric acid cycle

**Fig. 5:**
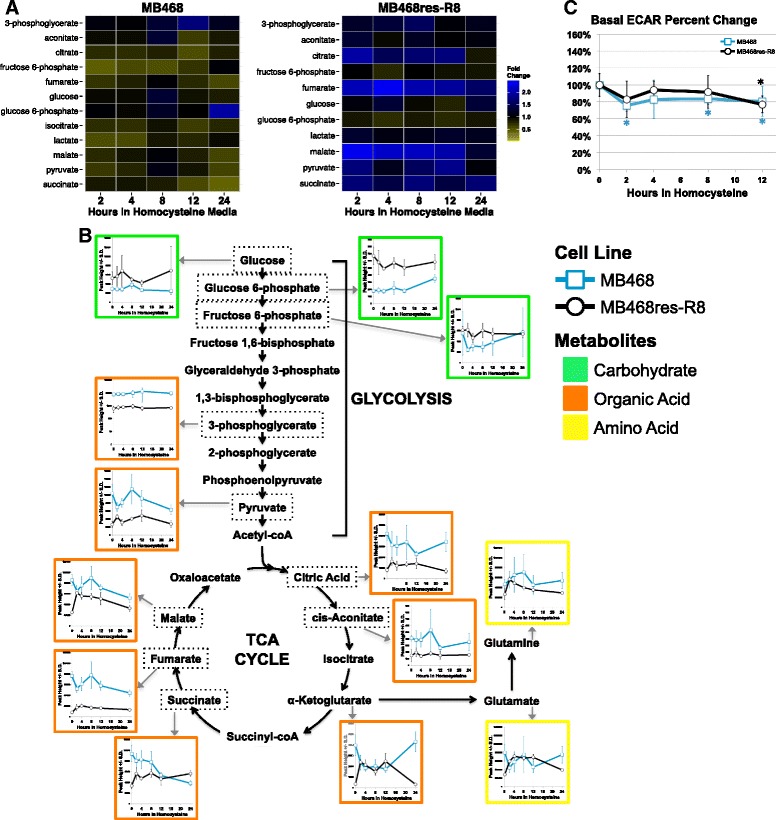
Metabolite changes in glycolysis and the TCA cycle associated with methionine stress. **a** MB468 and MB468res-R8 cells were cultured in Met+ or Met-Hcy+ media over the course of 24 h and analyzed by untargeted metabolomics as described for Fig. 4. Heatmaps represent fold changes of metabolites in glycolysis and the tricarboxylic acid (TCA) cycle as compared to the Met+, time-zero sample. Metabolites are color filled by fold change: *yellow* indicates a decrease in metabolite abundance and *blue* indicates an increase as compared to time-zero control. **b** Schematic of glycolysis and the TCA cycle. Metabolites with *gray arrows* are pointing to line graphs of the corresponding metabolite abundances in MB468 (*blue line* with *squares*) and MB468res-R8 (*black line* with *circles*) cells. Peak height reflecting relative metabolite abundance is indicated on the *y*-axis and hours in homocysteine are indicated on the *x*-axis. *Error bars* represent standard deviation. Metabolites included in the heatmaps (**a**) are outlined in a dashed box. **c** MB468 and MB468res-R8 cells cultured in Met+ media for 12 h or Met-Hcy+ media for 2, 4, 8, and 12 h were analyzed by the Seahorse Bioscience XF analyzer. MB468 (*blue line* with *squares*) and MB468res-R8 (*black line* with *circles*) basal extracellular acidification rates (ECAR) are shown normalized to the Met+-treated sample. *Error bars* represent normalized standard deviation, *n* = 4 replicates; statistical differences between Met+ media and Met-Hcy+ media treatments are indicated by *asterisk* where *p* ≤ 0.002

Despite major changes in other biochemical pathways, Met-Hcy+ media had minimal effects on the abundances of metabolites involved in glycolysis (Fig. 5a). Both MB468 and MB468res-R8 cells indicate only slightly decreased abundances in glucose and glucose 6-phosphate. More noticeable differences between the two cell lines were observed in fructose 6-phosphate and pyruvate. Unlike MB468res-R8, abundances of fructose 6-phosphate and pyruvate in MB468 indicated initial reductions post media switch followed by partial equilibration back toward baseline levels (Fig. 5b). Using the XF analyzer to further evaluate glycolysis, we treated MB468 and MB468res-R8 cells in Met+ media for 12 h or Met-Hcy+ media for 2, 4, 8, and 12 h and measured the ECAR, an indicator of glycolysis (Fig. 5c). Similar to many of the metabolite profiles, an initial decrease in glycolysis occurs within 2 h post media switch followed by partial recovery to baseline. MB468 cells decrease glycolysis at 2 h to 75 % baseline with an average partial recovery to 82 % over the next 10 h. MB468res-R8 cells follow a similar trend with a decrease in glycolysis to 83 % baseline at 2 h and average recovery to 87 % thereafter. These subtle changes in glycolysis suggest an early partitioning of glucose toward the pentose phosphate pathway for generation of NADPH (not detected) following the switch to Met-Hcy+ media.

Similar to our earlier observations of decreased mitochondrial respiration in Met-Hcy+ media (Fig. 2), the tricarboxylic acid (TCA)-related metabolites succinate, fumarate, and malate indicate general reductions in MB468 cells. In contrast, MB468res-R8 cells show a twofold increase in malate and fumarate with less striking increases in other TCA cycle components including citrate, succinate, and aconitate (Fig. 5b). The reduction in MB468-dependent TCA intermediates corroborates with decreased oxidative phosphorylation; however, the difference in response of MB468res-R8 may be a result of their lower respiration capacity and thus their reduced dependence on respiration in these cells.

### Homocysteine metabolism is redirected during HCY culturing

**Fig. 6:**
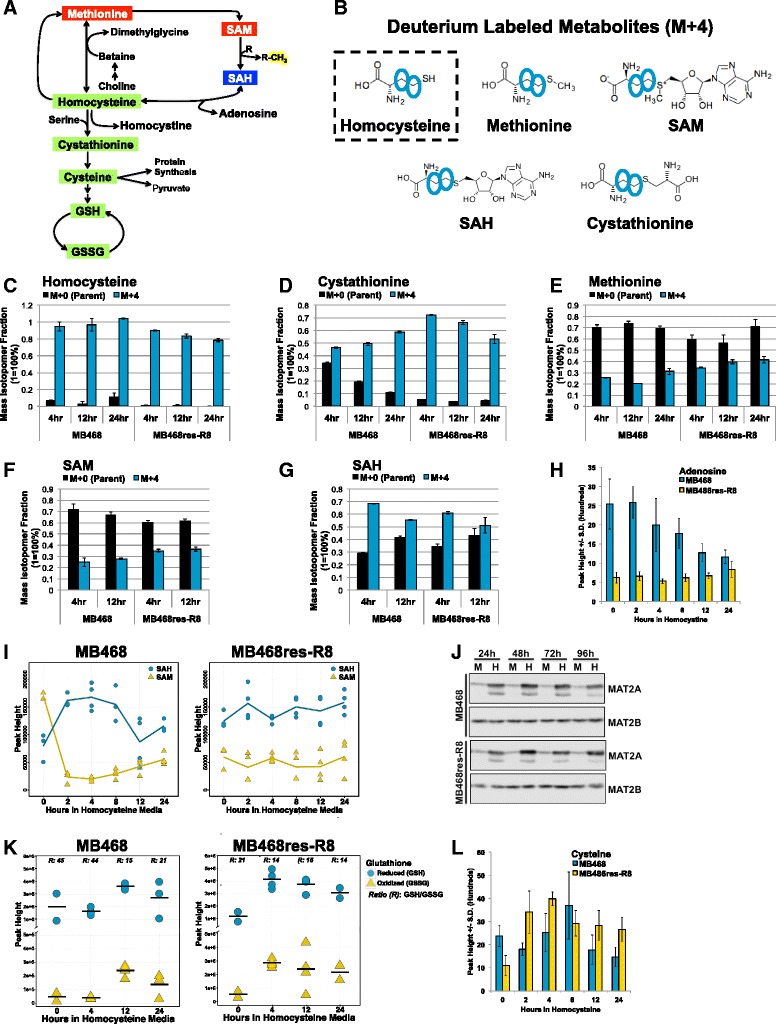
Homocysteine metabolism is redirected toward the transsulfuration pathway during methionine stress. **a** A schematic summarizing isotope tracer results. The majority of deuterium atoms labeled homocysteine and cystathionine, indicating a greater flux toward glutathione synthesis (*green*). Methionine and SAM abundances decreases (*red*) in both cell lines. SAH levels increase (*blue*) in MB468 cells only. The activated methyl group of SAM is highlighted with *yellow*. **b** Deuterated homocysteine (Hcy-_d4_(3,3,4,4), *dashed square*) was used to prepare Met-Hcy+ media for isotope tracer analysis. Metabolite structures with *blue circles* indicate the expected location of the deuterium atoms. Mass isotopomer distributions (MID) were calculated using the Fiehnlab MID Analyzer and validated by comparing spectra of enriched molecules to non-labeled spectra. MIDs for M+0 (*black*) non-labeled parent molecule and M+4 (*blue*), corresponding to a mass shift resulting from four deuterium atoms, are plotted for **c** homocysteine, **d** cystathionine, **e** methionine, **f** S-adenosylmethionine (SAM), and **g** S-adenosylhomocysteine (SAH) for MB468 and MB468res-R8 cells. **h** Adenosine levels for MB468 (*blue*) and MB468res-R8 (*yellow*) cells were measured by GCTOFMS. *Error bars* represent standard deviation. **i** SAM (*yellow triangles*) and SAH (*blue circles*) levels were measured by HILIC-UHPLC-qTOFMS in MB468 and MB468res-R8 cells. Scatter plots represent peak heights for individual replicates. The overlaid line graph (SAM, *yellow*, SAH, *blue*) indicates average peak heights. A minimum of two replicates were measured per sample. **j** MB468 and MB468res-R8 cells cultured in Met+ (“M”) or Met-Hcy+ (“H”) media were analyzed for methionine adenosyltransferase 2A and 2B (MAT2A, MAT2B) by immunoblot. **k** Glutathione pools are shown for MB468 and MB468res-R8 cells. Reduced (GSH, *blue circles*) and oxidized (GSSG, *yellow triangles*) glutathione species are plotted for each time point post Met-Hcy+ media switch; *horizontal bars* indicate averages for each replica set. Ratios of GSH/GSSG are listed above each time point. **l** Cysteine levels for MB468 (*blue*) and MB468res-R8 (*yellow*) cells were measured by GCTOFMS. *Error bars* represent standard deviation

The changes in steady state levels of metabolites are informative and indicate a significant rewiring of metabolic pathways in response to methionine stress and highlight different metabolic responses in methionine-dependent and methionine-independent cell lines. However, interpretations of steady state level changes are complicated by reversibility of reactions, such that pathway activities or flux changes can only be inferred in some situations. The methionine dependence of cancer is closely linked to the availability of SAM as a co-factor for methylation, which is also illustrated by the complete suppression of growth inhibition of MB468 cells cultured in Met-Hcy+ supplemented with SAM [10]. We therefore asked how homocysteine is used in cells over time with particular focus on remethylation and transsulfuration (Fig. 6a). To this end, we used deuterium-labeled homocysteine (_d4_Hcy = Hcy-_d4_(3,3,4,4)) to replace methionine in the cell growth media (Fig. 6b). Both MB468 and MB468res-R8 cells accumulated _d4_Hcy to about the same extent confirming that uptake of homocysteine cannot account for methionine stress sensitivity (Fig. 6c). Methionine flux analyses have shown that generally homocysteine remethylation and entry into the transsulfuration pathway each accounts for approximately 50 % [40, 41]. Surprisingly, post Met-_d4_Hcy+ media switch, the majority of homocysteine metabolism in both MB468 and MB468res-R8 is predominantly directed toward the transsulfuration pathway as indicated by the almost complete dominance of deuterated cystathionine (transsulfuration) after 24 h and underrepresentation of deuterated methionine (Fig. 6d, e). However, this effect was significantly delayed in MB468 cells. The methionine stress resistant MB468res-R8 cells showed a complete shift to deuterated cystathionine within 4 h of the media switch, whereas methionine stress sensitive MB468 cells required at least 24 h in Met-_d4_Hcy+ medium to show a comparable transition to deuterated cystathionine. These studies also revealed that MB468res-R8 cells use homocysteine to remethylate an average of 13 % more methionine molecules as compared to MB468 cells, a difference that is reflected in labeled SAM levels as well (Fig. 6f).

It is interesting to note that while MB468res-R8 cells process homocysteine forward through the remethylation pathway, MB468 cells process an average of 6 % more homocysteine through the reversible hydrolytic activity of S-adenosylhomocysteine hydrolase (Fig. 6g). SAH is the by-product of all biological transmethylation reactions requiring SAM and is also generated from homocysteine and adenosine. This reverse processing of homocysteine in MB468 cells may partially contribute to the decrease in adenosine abundance (Fig. 6h). Comparing the relative concentration of SAM and SAH can indicate the methylation potential of the cell. However, it has been shown that a reduced ratio bares little consequence without a corresponding increase in SAH [42, 43]. In both MB468 and MB468res-R8 cells, SAM and SAH were measured over a 24-h period in Met-Hcy+ media (Fig. 6i). For both cell lines, the SAM to SAH ratio decreased within 2 h of the media switch. In MB468 cells, this decrease in SAM to SAH is a result of a simultaneous decrease in SAM to less than one fifth starting quantity and increase in SAH to 1.5 times the starting quantity. After 12 h, however, both SAH and SAM begin to recover toward starting levels. In contrast, the SAM to SAH decrease observed in MB468res-R8 cells is a result of SAM levels declining to half the starting quantity with minimal perturbations in SAH. Considering SAM levels are twofold greater in MB468 cells in Met+ media as compared to MB468res-R8 cells, the consequences of an altered SAM to SAH ratio and elevated SAH levels in MB468 cells on the methylation potential of the cell may play an important role in methionine sensitivity. Clearly, both cell lines experience limited methylation potential as indicated by feedback up-regulation of methionine adenosyltransferase (MAT), the enzyme that produces SAM from methionine and ATP (Fig. 6j). Although labeled methionine levels are higher in MB468res-R8 cells, in both cell lines, it appears that the catalytic MAT2A subunit is, for unknown reasons, unable to use the available methionine for SAM synthesis and therefore protein levels increase to compensate this deficiency. Interestingly, MAT2B, the regulatory subunit of the enzyme, is unaffected by methionine perturbations.

### Homocysteine and its relationship to ROS, mitochondria, and oxidative stress

Transsulfuration is critical to maintain the cellular redox balance as this metabolic pathway leads directly to glutathione synthesis (Fig. 6a). Interestingly, signaling pathways in yeast that are activated by methionine or SAM limitation induce a massive increase in glutathione synthesis suggesting a common physiological response to methionine stress in yeast and mammals [44–47]. Accordingly, the redirection of homocysteine metabolism to the transsulfuration pathway suggests the activation of a protective mechanism in response to Met-Hcy+ media. Under normal culturing conditions, the majority of cellular glutathione exists in its reduced form (GSH). As an antioxidant, glutathione protects the cell from oxidative stress by reducing the disulfide bonds of cytoplasmic proteins and in the process is oxidized to form glutathione disulfide (GSSG) [48]. Interestingly, in Met+ media the abundance of GSH is twice as much in MB468 as compared to MB468res-R8 despite similar levels of GSSG (Fig. 6k). After culturing in Met-Hcy+ media, both cell lines experience oxidative stress as indicated by increasing levels of GSSG and a steep increase in GSH, presumably to combat oxidative pressure. The timing of this transition to the transsulfuration pathway and glutathione synthesis occurred within 4 h post media switch in MB468res-R8 cells and by 12 h in MB468 cells. This timing mirrors the increases of labeled cystathionine in both cell lines (Fig. 6d), supporting the hypothesis that redirection of homocysteine metabolism to transsulfuration is due to the cellular need to balance redox potentials. Cysteine abundances could not be analyzed by stable isotope tracer studies because metabolic processing of cystathionine results in an unlabeled molecule of cysteine and a deuterium-labeled alpha-ketobutyrate molecule. However, using an untargeted approach, cysteine levels indicate a similar increase as compared to both glutathione and cystathionine with peak abundances at 8 h in MB468 and 4 h in MB468res-R8 cells post media switch (Fig. 6l).

The oxidative stress associated with Met-Hcy+ media is not unexpected as studies on atherosclerosis have identified a relationship between elevated homocysteine levels in plasma and oxidative stress in tissue [49]. The observed stress may be attributed to auto-oxidation of homocysteine, which generates hydrogen peroxide and reactive oxygen species (ROS). Alternatively, the source of stress may be related to inhibition of ATP synthesis in the mitochondria. N-methyl-d-aspartate (NMDA) receptors are glutamate and glycine-activated calcium channels that are largely found in neurons. Recent studies have identified active NMDA receptors in human neuroblastoma cells, small-cell lung cancer cells, and breast cancer cells and tumors, but not normal tissues [50, 51]. Free and reduced homocysteine molecules can bind to NMDA receptors at the glutamate binding location and activate an influx of calcium ions resulting in reduced ATP synthesis, reduced oxidative phosphorylation, and an accumulation of ROS [52]. Although this is a suggested mechanism for Met-Hcy+-induced oxidative stress, the loss of oxidative phosphorylation activity is evident in both cell lines (Fig. 2) and the response to counter an oxidative insult by increasing glutathione abundance supports this scenario (Fig. 6k).

**Fig. 7:**
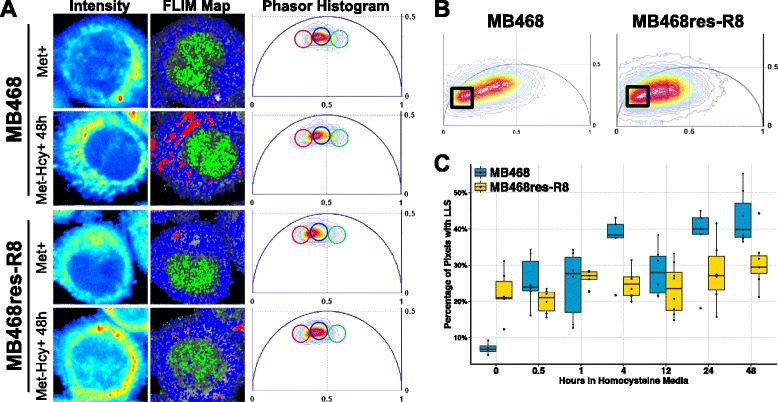
Methionine stress induces an increase in oxidative stress. **a** Two-photon fluorescence intensity images of individual MB468 and MB468res-R8 cells in Met+ or Met-Hcy+ media (48 h). Pixels within three groups in the phasor histogram have been isolated and colored accordingly in the FLIM map image. Higher free/bound NADH ratios are highlighted in *green*, indicating a more reduced state in the nucleus. Lower free/bound NADH ratios are highlighted in *blue*, indicating higher ratios of oxidative phosphorylation versus glycolysis in the mitochondria/cytoplasm. Long lifetime species (LLS) are highlighted in *red*, indicating the presence of oxidized lipids in the cell. **b** Phasor histograms of MB468 and MB468res-R8 cells. The *black box* selects the phasors with LLS signature. **c** A boxplot representing the percentage of pixels with the LLS (oxidized lipids) signature in MB468 (*blue*) and MB468res-R8 (*yellow*) in in Met-Hcy+ over a 48 h time course

In addition to reduced glutathione ratios, the presence of oxidized lipids becomes more prevalent upon Met-Hcy+ media switch. The FLIM approach can effectively visualize and measure oxidized lipids at the single cell and sub-cellular level. Oxidized lipids are detected by a long fluorescence lifetime (LLS) and serve as biomarkers for oxidative stress (Fig. 7a) [53]. To quantify the LLS fraction in each field, we used phasor cluster analysis of MB468 and MB468res-R8 cells cultured in Met-Hcy+ media over the course of 48 h (Fig. 7b). In normal Met+ culturing conditions, the average fraction of LLS in MB468 cells is 7 and 22 % in MB468res-R8 cells (Fig. 7c). Immediately after media switch to Met-Hcy+, the fraction of LLS increases in MB468 to 25 % at 30 min and continues to increase over time to an average of 36 % at 48 h. In contrast, MB468res-R8 cells respond to the media shift with a 5 % average increase to 27 % LLS after 48 h. It is interesting to note the higher level of oxidized lipids in MB468res-R8 cells in Met+ as compared to MB468 cells and yet the striking increase observed in the MB468 cells over time in Met-Hcy+. The biological functions of these LLS species are not known but are speculated to serve a protective function that shields cells from oxidative damage due to the sequestering of oxidized lipid in vesicles.

## Conclusions

In conclusion, the present study reveals dynamic changes in the metabolic phenotype associated with methionine dependency of breast cancer and further highlights the importance of SAM as a major regulator of tumorigenesis. Importantly, using a labeled precursor, we discovered that in both MB468 and MB468res-R8 cell types, homocysteine does not significantly contribute to formation of methionine and ultimately S-adenosylmethionine. Our study indicates that SAM metabolism, which is required for all methylation reactions, depends on availability of methionine in the cancer cell lines investigated and cannot be substituted by homocysteine-forming pathways. Moreover, these findings shed light on the metabolic reprogramming, which accompanies methionine stress beyond methylation potential. Such knowledge is instrumental for elucidating novel targets of therapeutic intervention, which can be exploited in future studies. Given the importance of SAM in lipid biogenesis and our observed changes in LLS, assessing the impact of methionine stress on lipid composition and synthesis is of considerable interest and warrants further investigation.

## Abbreviations

ECAR: extracellular acidification rate
FCCP: carbonyl cyanide-4-phenylhydrazone
FLIM: fluorescence lifetime imaging microscopy
GCTOFMS: gas chromatography time-of-flight mass spectrometry
GSH: reduced glutathione
GSSG: glutathione disulfide, oxidized glutathione
HILIC-UHPLC-qTOFMS: hydrophilic interaction liquid chromatography ultra high-pressure liquid chromatography accurate mass quadrupole time-of-flight mass spectrometry
KCN: potassium cyanide
LLS: long lifetime species
MAT2A: methionine adenosyltransferase II, alpha
MAT2B: methionine adenosyltransferase II, beta
MB468: MDA-MB-468 breast cancer cell line
MB468res-R8: MDA-MB-468res-R8 methionine stress resistant breast cancer derived cell line
Met+: methionine media
Met-Hcy+: homocysteine media without methionine
MID: mass isotopomer distribution
MTA: 5'-deoxy-5'-methylthioadenosine
NADH: nicotinamide adenine dinucleotide
OCR: oxygen consumption rate
SAH: S-adenosylhomocysteine
SAM: S-adenosylmethionine
TCA: tricarboxylic acid

## References

[1] Hanahan D, Weinberg RA (2011). Hallmarks of cancer: the next generation. Cell.

[2] Sugimura T, Birnbaum SM, Winitz M, Greenstein JP (1959). Quantitative nutritional studies with water-soluble, chemically defined diets. VIII. The forced feeding of diets each lacking in one essential amino acid. Arch Biochem Biophys.

[3] Halpern BC, Clark BR, Hardy DN, Halpern RM, Smith RA (1974). The effect of replacement of methionine by homocystine on survival of malignant and normal adult mammalian cells in culture. Proc Natl Acad Sci U S A.

[4] Sperber H, Mathieu J, Wang Y, Ferreccio A, Hesson J, Xu Z, Fisher KA, Devi A, Detraux D, Gu H, Battle SL, Showalter M, Valensisi C, Bielas JH, Ericson NG, Margaretha L, Robitaille AM, Margineantu D, Fiehn O, Hockenbery D, Blau CA, Raftery D, Margolin A, Hawkins RD, Moon RT, Ware CB, Ruohola-Baker H. The metabolome regulates the epigenetic landscape during naive to primed human embryonic stem cell transition. Nat Cell Biol*.* 2015;17(12):1523–35. 10.1038/ncb3264 PMC4662931 26571212

[5] Hu X, Washington S, Verderame MF, Demers LM, Mauger D, Manni A (2004). Biological activity of the S-adenosylmethionine decarboxylase inhibitor SAM486A in human breast cancer cells in vitro and in vivo. Int J Oncol.

[6] Tang B, Li YN, Kruger WD (2000). Defects in methylthioadenosine phosphorylase are associated with but not responsible for methionine-dependent tumor cell growth. Cancer Res.

[7] de la Haba G, Cantoni GL (1958). of S-Adenosyl-L-homocysteine and homocysteine*. Enzyme.

[8] Finkelstein JD, Mudd SH (1967). Trans-sulfuration in mammals.

[9] Coalson DW, Mecham JO, Stern PH, Hoffman RM. Reduced availability of endogenously synthesized methionine for S-adenosylmethionine formation in methionine-dependent cancer cells. pnas.org. 79(14):4248-51. 10.1073/pnas.79.14.4248 PMC346647 6289297

[10] Booher K, Lin DW, Borrego SL, Kaiser P (2012). Downregulation of Cdc6 and pre-replication complexes in response to methionine stress in breast cancer cells. Cell Cycle.

[11] Lin DW, Chung BP, Kaiser P (2013). S-adenosylmethionine limitation induces p38 mitogen-activated protein kinase and triggers cell cycle arrest in G1. J Cell Sci.

[12] Giardiello FM, Hamilton SR, Hylind LM, Yang VW, Tamez P, Casero RA (1998). Ornithine decarboxylase and polyamines in familial adenomatous polyposis. Clin Cancer Res.

[13] Pegg AE, McCann PP. Polyamine metabolism and function. Am J Physiol. 1982;243(5):C212–21. 10.1152/ajpcell.1982.243.5.C212 6814260

[14] Wikoff WR, Hanash S, DeFelice B, Miyamoto S, Barnett M, Zhao Y, Goodman G, Feng Z, Gandara D, Fiehn O, Taguchi A. Diacetylspermine is a novel prediagnostic serum biomarker for non-small-cell lung cancer and has additive performance with pro-surfactant protein B. JClin Oncol. 2015;33(33):3880-6. 10.1200/JCO.2015.61.7779 PMC4652011 26282655

[15] Johnson CH, Dejea CM, Edler D, Hoang LT, Santidrian AF, Felding BH, Ivanisevic J, Cho K, Wick EC, Hechenbleikner EM, Uritboonthai W, Goetz L, Casero RA, Pardoll DM, White JR, Patti GJ, Sears CL, Siuzdak G (2015). Metabolism links bacterial biofilms and colon carcinogenesis. Cell Metab.

[16] Seidenfeld J, Block AL, Komar KA, Naujokas MF (1986). Altered cell cycle phase distributions in cultured human carcinoma cells partially depleted of polyamines by treatment with difluoromethylornithine. Cancer Res.

[17] Hoffman RM, Jacobsen SJ, Erbe RW (1979). Reversion to methionine independence in simian virus 40-transformed human and malignant rat fibroblasts is associated with altered ploidy and altered properties of transformation. Proc Natl Acad Sci U S A.

[18] Fan TW. The handbook of metabolomics. 2012;17.

[19] Stringari C, Cinquin A, Cinquin O, Digman MA, Donovan PJ, Gratton E (2011). Phasor approach to fluorescence lifetime microscopy distinguishes different metabolic states of germ cells in a live tissue. Proc Natl Acad Sci U S A.

[20] Stringari C, Nourse JL, Flanagan LA, Gratton E (2012). Phasor fluorescence lifetime microscopy of free and protein-bound NADH reveals neural stem cell differentiation potential. PLoS ONE.

[21] Scholz M, Fiehn O (2007). Setup X—a public study design database for metabolomic projects. Biocomput 2007 - Proc Pacific Symp.

[22] Fiehn O, Wohlgemuth G, Scholz M, Kind T, Lee DY, Lu Y, Moon S, Nikolau B (2008). Quality control for plant metabolomics: reporting MSI-compliant studies. Plant J.

[23] Fiehn O, Wohlgemuth G, Sholz M (2005). Setup and annotation of metabolomic experiments by integrating biological and mass spectrometric metadata. Dils.

[24] Kind T, Tolstikov V, Fiehn O, Weiss RH (2007). A comprehensive urinary metabolomic approach for identifying kidney cancer. Anal Biochem.

[25] Kind T, Liu K-H, Lee DY, DeFelice B, Meissen JK, Fiehn O (2013). LipidBlast in silico tandem mass spectrometry database for lipid identification. Nat Methods.

[26] Hiller K, Metallo CM, Kelleher JK, Stephanopoulos G (2010). Nontargeted elucidation of metabolic pathways using stable-isotope tracers and mass spectrometry. Anal Chem.

[27] Jennings ME, Matthews DE (2005). Determination of complex isotopomer patterns in isotopically labeled compounds by mass spectrometry. Anal Chem.

[28] Kreis W, Goodenow M (1978). Methionine requirement and replacement by homocysteine in tissue cultures of selected rodent and human malignant and normal cells. Cancer Res.

[29] Cavuoto P, Fenech MF (2012). A review of methionine dependency and the role of methionine restriction in cancer growth control and life-span extension. Cancer Treat Rev.

[30] Hoffman RM, Jacobsen SJ, Erbe RW (1978). Reversion to methionine independence by malignant rat and SV40-transformed human fibroblasts. Biochem Biophys Res Commun.

[31] Cassingena R, Lafarge-Frayssinet C, Painchault V, Estrade S, Nardeux P, Frayssinet C (1990). Methionine-independence, tumorigenicity and oncogene expression of rat hepatocarcinoma cells. Biol Cell.

[32] Heikal AA (2010). Intracellular coenzymes as natural biomarkers for metabolic activities and mitochondrial anomalies. Biomark Med.

[33] Bird DK, Yan L, Vrotsos KM, Eliceiri KW, Vaughan EM, Keely PJ, White JG, Ramanujam N (2005). Metabolic mapping of MCF10A human breast cells via multiphoton fluorescence lifetime imaging of the coenzyme NADH. Cancer Res.

[34] Lakowicz JR, Szmacinski H, Nowaczyk K, Johnson ML (1992). Fluorescence lifetime imaging of free and protein-bound NADH. Proc Natl Acad Sci U S A.

[35] Stringari C, Edwards RA, Pate KT, Waterman ML, Donovan PJ, Gratton E. Metabolic trajectory of cellular differentiation in small intestine by phasor fluorescence lifetime microscopy of NADH. Sci Rep*.* 2012;2. 10.1038/srep00568 PMC3416911 22891156

[36] Pate KT, Stringari C, Sprowl-Tanio S, Wang K, TeSlaa T, Hoverter NP, McQuade MM, Garner C, Digman MA, Teitell MA, Edwards RA, Gratton E, Waterman ML. Wnt signaling directs a metabolic program of glycolysis and angiogenesis in colon cancer. EMBO J*.* 2014. 10.15252/embj.201488598 PMC4194089 24825347

[37] Baldi P, Long AD (2001). A Bayesian framework for the analysis of microarray expression data: regularized t-test and statistical inferences of gene changes. Bioinformatics.

[38] Kayala MA, Baldi P (2012). Cyber-T web server: differential analysis of high-throughput data. Nucleic Acids Res.

[39] Chagoyen M, Pazos F (2011). MBRole: enrichment analysis of metabolomic data. Bioinformatics.

[40] Turner MA, Yang X, Yin D, Kuczera K, Borchardt RT, Howell PL (2000). Structure and function of S-adenosylhomocysteine hydrolase. Cell Biochem Biophys.

[41] Storch KJ, Wagner DA, Burke JF, Young VR (1990). [1-13C; methyl-2H3]methionine kinetics in humans: methionine conservation and cystine sparing. Am J Physiol.

[42] Hoffman DR, Marion DW, Cornatzer WE, Duerre JA (1980). S-adenosylmethionine and S-adenosylhomocysteine metabolism in isolated rat liver. J Biol Chem.

[43] Caudill MA, Wang JC, Melnyk S, Pogribny IP, Jernigan S, Collins MD, Santos-Guzman J, Swendseid ME, Cogger EA, James SJ (2001). Intracellular S-adenosylhomocysteine concentrations predict global DNA hypomethylation in tissues of methyl-deficient cystathionine beta-synthase heterozygous mice. J Nutr.

[44] Wheeler GL, Quinn KA, Perrone G, Dawes IW, Grant CM (2002). Glutathione regulates the expression of gamma-glutamylcysteine synthetase via the Met4 transcription factor. Mol Microbiol.

[45] Wheeler GL, Trotter EW, Dawes IW, Grant CM (2003). Coupling of the transcriptional regulation of glutathione biosynthesis to the availability of glutathione and methionine via the Met4 and Yap1 transcription factors. J Biol Chem.

[46] Kaiser P, Su N-Y, Yen JL, Ouni I, Flick K (2006). The yeast ubiquitin ligase SCFMet30: connecting environmental and intracellular conditions to cell division. Cell Div.

[47] Lee TA, Jorgensen P, Bognar AL, Peyraud C, Thomas D, Tyers M (2010). Dissection of combinatorial control by the Met4 transcriptional complex. Mol Biol Cell.

[48] Liou G-Y, Storz P (2010). Reactive oxygen species in cancer. Free Radic Res.

[49] Olszewski J, McCully KS (1993). Homocysteine metabolism adn the oxidative modification of proteins and lipids. Free Radic Biol Med.

[50] North WG, Gao G, Jensen A, Memoli VA, Du J (2010). NMDA receptors are expressed by small-cell lung cancer and are potential targets for effective treatment. Clin Pharmacol Adv Appl.

[51] North WG, Gao G, Memoli VA, Pang RH, Lynch L (2010). Breast cancer expresses functional NMDA receptors. Breast Cancer Res Treat.

[52] McCully KS (2009). Chemical pathology of homocysteine. IV. Excitotoxicity, oxidative stress, endothelial dysfunction, and inflammation. Ann Clin Lab Sci.

[53] Datta R, Alfonso-García A, Cinco R, Gratton E (2015). Fluorescence lifetime imaging of endogenous biomarker of oxidative stress. Sci Rep.

